# Longitudinal Variability of Phosphorus Fractions in Sediments of a Canyon Reservoir Due to Cascade Dam Construction: A Case Study in Lancang River, China

**DOI:** 10.1371/journal.pone.0083329

**Published:** 2013-12-26

**Authors:** Qi Liu, Shiliang Liu, Haidi Zhao, Li Deng, Cong Wang, Qinghe Zhao, Shikui Dong

**Affiliations:** School of Environment, State Key Laboratory of Water Environment Simulation, Beijing Normal University, Beijing, China; University of Shiga Prefecture, Japan

## Abstract

Dam construction causes the accumulation of phosphorus in the sediments of reservoirs and increases the release rate of internal phosphorus (P) loading. This study investigated the longitudinal variability of phosphorus fractions in sediments and the relationship between the contents of phosphorus fractions and its influencing factors of the Manwan Reservoir, Lancang River, Yunnan Province, China. Five sedimentary phosphorus fractions were quantified separately: loosely bound P (ex-P); reductant soluble P (BD-P); metal oxide-bound P (NaOH-P); calcium-bound P (HCl-P), and residual-P. The results showed that the total phosphorus contents ranged from 623 to 899 µg/g and were correlated positively with iron content in the sediments of the reservoir. The rank order of P fractions in sediments of the mainstream was HCl-P>NaOH-P>residual-P>BD-P>ex-P, while it was residual-P>HCl-P>NaOH-P>BD-P>ex-P in those of the tributaries. The contents of bio-available phosphorus in the tributaries, including ex-P, BD-P and NaOH-P, were significantly lower than those in the mainstream. The contents of ex-P, BD-P, NaOH-P showed a similar increasing trend from the tail to the head of the Manwan Reservoir, which contributed to the relatively higher content of bio-available phosphorus, and represents a high bio-available phosphorus releasing risk within a distance of 10 km from Manwan Dam. Correlation and redundancy analyses showed that distance to Manwan Dam and the silt/clay fraction of sediments were related closely to the spatial variation of bio-available phosphorus.

## Introduction

Dam construction can change rivers’ configurations and flow regimes [1], [2], [3], which will have conspicuous direct effects on nutrient loading in the rivers [4], [5]. For example, after the closure of the Three-Gorges Dam in China, the nutrient concentrations and ratios in the water declined dramatically during the high flood season [4]. In the upper stream of the Yellow River, nutrient pollutants varied greatly because of the construction of cascade dams [6]. Dams reduce the transportation of nutrients to marine waters, which indicates that more nutrients settle in the sediments [5].

Among different nutrient pollutants, phosphorus (P) has attracted much attention in sediment research as a key nutrient for phytoplankton growth, controlling the primary productivity of reservoirs [7], [8], [9], [10], [11]. When external loading of P occurs, such as from an increase in the drainage of intensively cultivated areas and from sewage, the rate of accumulation of P in the sediments exceeds its ability to release P into water, so sediments act as P sinks. While the external P is reduced, the sediments still release P into the water, which is called internal phosphorus (P) loading [8], [9], [11], [12], [13], [14], [15], [16]. Previous research has found that, even when the external phosphorus loading was reduced, P concentrations in lakes either did not change or decreased slightly because of internal phosphorus loading [17], [18]. Further, P released by internal P loading contributes to the pool of the P used easily by algae in sediments [19]. Hence, internal P loading has been of great concern in recent years because it can be a potential hazard to aquatic ecosystems [20]. For example, a study in Taihu Lake in China indicated that more than 50% of the inorganic P could be released into the water and used by algae under certain conditions [21]. Although there are several forms of P in sediments, not all of them are released easily from the sediments into the water [22], as this depends on the characteristics of the sediment, environmental factors and the concentration of P in water as well [8], [14], [22], [23], [24]. Studies have shown that grain size distribution of the sediments influences the element composition in the sediments, including metal and nutrient contents [25], [26], [27]. Stone and English (1993) [19] found that fine grained sediment had a different relationship with different P fractions. Further, in river systems influenced by hydropower dams, the spatial grain size distribution of the sediments might be affected by the formative reservoirs and prolonged water renewal time, which eventually has an effect on the heterogeneity of different P fractions in the sediments. Recently, determining P fractions in sediments and the releasing capacity of P fractions from the sediments to the water has been studied and reviewed extensively [12], [28], [29], [30], [31]. However, the study of the mechanism of the longitudinal variability of phosphorus fractions in the sediments of canyon reservoirs affected by cascade dams is insufficient and needs further research.

Studies have shown that, in the Lower Mekong River mainstream, countries through which the Mekong flows are currently threatened by accelerated eutrophication caused by both hydropower development and climate change [32]. Moreover, the Mekong River Commission (MRC) Water Quality Report (2008) noted that almost one-third of the total P in the Lower Mekong Basin exists as soluble orthophosphate (PO_4_-P), which indicated that approximately two-thirds of the total P loading was associated with sediments. In the Upper Mekong River, the external loading of P is lower than that of the Lower Mekong River. Therefore, even slight changes in sediment conditions are likely to affect the P concentration of the water column [33]. However, there are few studies regarding the nutrient condition of the sediments in the Upper Mekong section in China (Lancang River), where a chain of fourteen cascade hydroelectric dams have been planned since the early 1980s, with some completed or currently under construction. Further, human activities are becoming more and more intense, with industrialization and fertilization causing an increased P loading in the river. The Manwan Dam was the first dam constructed in the cascade development project along the Lancang River mainstream. After the Manwan Reservoir began operation, siltation led to increasing concern from researchers and the public because it had increased the elevation of the reservoir bottom by as much as 30 m caused by dam construction [34]. Studies have shown that there was great spatial and temporal variation of heavy metals in the sediments of the Manwan Reservoir, and some metals, such as As, Cd, Cr, Cu, Pb and Zn, have reached contamination levels [35], [36]. Further, the study of Zhao, et al. [37], revealed that the potential ecological risk (RI) of multiple heavy metals was related to the grain size of sediments and correlated negatively with the distance from Manwan Dam, a valuable finding for understanding heavy metal contamination in a canyon reservoir. However, the possible spatial variation of P fractions in sediments remains unknown, and this information is useful in understanding the dynamic of trophic conditions of the Manwan Reservoir induced by dam construction.

The aims of our study were to (1) investigate the spatial variation of different P fractions in river sediments of the mainstream and the tributaries of the Manwan Reservoir; (2) estimate the contents of bio-available P in the sediments of Manwan Reservoir, and (3) explore the relationship among P fractions and influencing factors, including metals and sediment grain size.

## Materials and Methods

### Study Site

**Figure 1:**
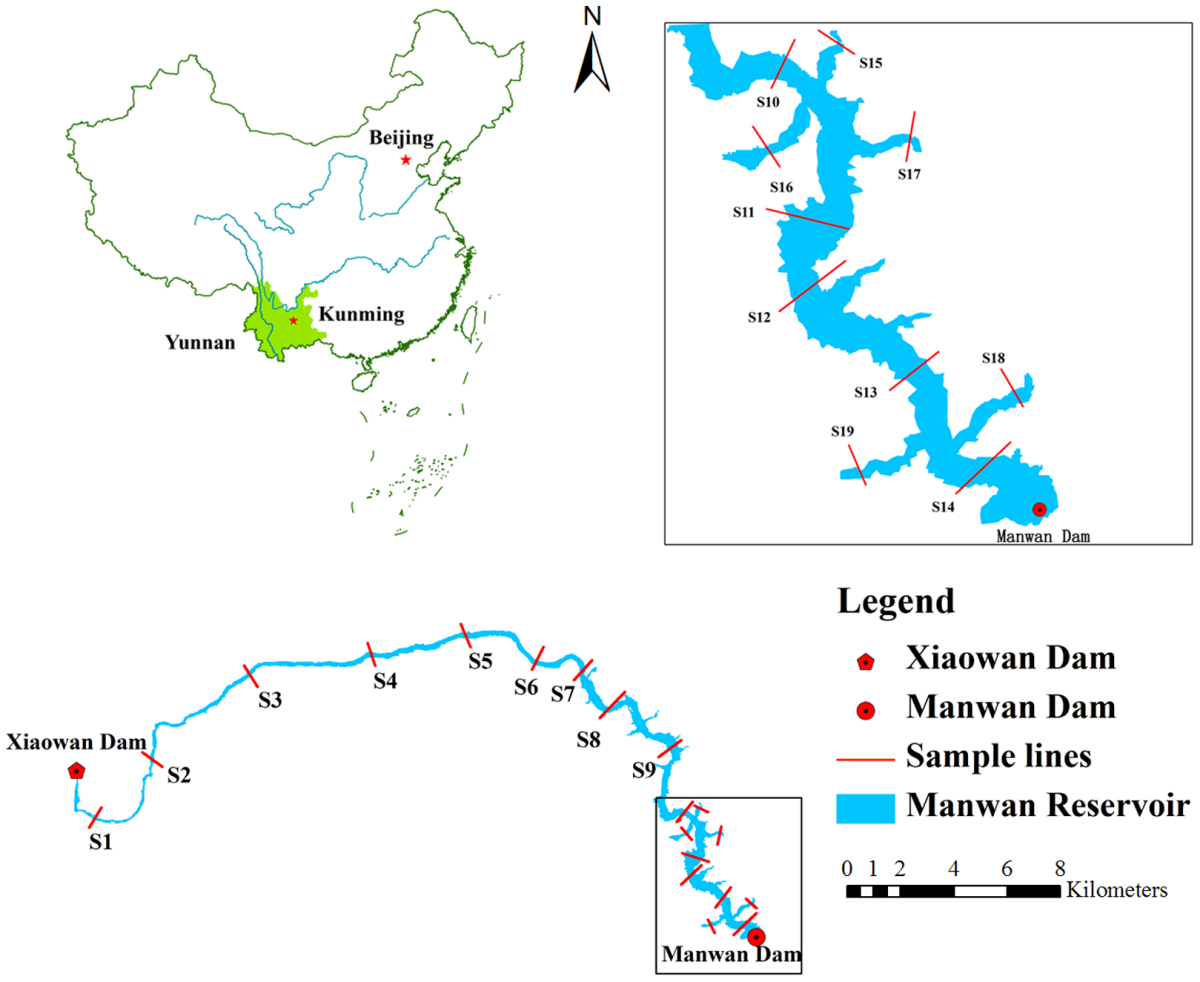
Location of the Manwan Reservoir and the 19 cross-sectional sediment samples, Yunnan Province, China.

The Mekong River is the largest international river in Asia, flowing through seven climatic zones and five countries; it is considered to be one of the most important cradles of human civilization in Southeast Asia [38], [39]. The Upper Mekong Section in China (Lancang River), with almost 91% of the drop in elevation of the Mekong River, produces plentiful hydraulic resources [40], [41], [42]. To date, fourteen cascade dams have been planned there since the early 1980s [35], four of which have been constructed (Xiaowan, Manwan, Dachaoshan and Jinghong) in Yunnan Province. The Manwan Dam, completed in 1993, is the first multimillion kilowatt hydropower station in Yunnan Province [39], [42]. The dam is 418 m long and 132 m high with a backwater of 70 km near the Xiaowan Dam. The Manwan Reservoir was a canyon reservoir located in a gorge flanked by high mountains, most of whose peaks are higher than 2,200 m above sea level, and deep valleys with a gradient ratio over 15% [36] (Fig. 1). The area of Manwan Reservoir is 23.6 km^2^, and the width of the water surface is, on average, 337.1 m, 2.8 times larger in area and 2 times wider than it was before dam construction. The total reservoir capacity is 1,060×10^6^ m^3^, with a normal water level of 994 m; the effective capacity is 257×10^6^ m^3^ depending upon seasonal discharge regulation [42].

### Sediment Sample Collection

Manwan Dam was constructed and is managed by China Huaneng Group Corporation, a state-owned enterprise. The corporation gave us permission to conduct this field study, which did not involve any endangered or protected species. It was environmentally neutral and did not threaten the welfare of any species or that of the local population. Therefore, it was not related to ethical issues and no specific permissions were required for such activities.

In June, 2012, nineteen surface (15 cm) sediment samples were collected, using cable operated sediment samplers (Van Veen grabs) to investigate the spatial variation in P fractions in the sediments of the Manwan Reservoir. Fourteen cross-sectional samples (S1–S14) were selected to examine the variations in the mainstream of the reservoir, and the remaining five cross-sectional samples (S15–S19) were located at the major tributaries of the reservoir near the dam (Fig. 1). Each sample in a cross-section was a mixture of three sampling sites: the left, middle and right of each section. Both the right and left sampling sites were selected at the same distance to the shore. All of the samples were placed in sealed plastic bags and maintained at 4°C until analysis. After transportation to the laboratory, the samples were kept frozen, and before the analysis, were freeze-dried and ground until all of the particles passed through a 2 mm nylon sieve after removal of the coarse debris [27], [43], [44].

### Analytical Methods

For P fractionation of the sediments from the reservoir, we used the chemical sequential extraction method of Psenner et al. [45], slightly modified by Hupfer et al. [46]. This method fractionates the phosphorus of the sediments into four fractions–loosely bound P (ex-P), reductant soluble P (BD-P), metal oxide-bound P (NaOH-P), calcium-bound P (HCl-P; Table 1) and residual-P–which was the difference between total phosphorus and the four P fractions extracted. All procedures were carried out in triplicate to yield reliable results.

**Table 1:** Extraction procedure used in this work.

step	sequential extraction method	P fraction
1	1 g sediments added to 25 ml 1 M NH_4_Cl at pH = 7 shaken for 4 h.	ex-P
2	Residual sample added to 0.11 M Na_2_S_2_O_4_/NaHCO_3_ * shaken for 1 h at 40°C.	BD-P
3	Residual sample added to 0.1 M NaOH shaken for 16 h.	NaOH-P
4	Residual sample added to 0.5 M HCl shaken for 16 h	HCl-P

Both Na_2_S_2_O_4_ and NaHCO_3_ were the same concentration.

The extracts in every step were centrifuged at 4500 r/min for 20 minutes, and the soluble reactive phosphorus (SRP) in each fraction was determined by the molybdenum blue/ascorbic acid method (APHA, 1985). For the NaOH extracts, the supernatants were filtered through a 0.45-µm poly-amide filter.

The concentration of total phosphorus was determined by ICP-AES after acid digestion of the freeze-dried samples. Residual phosphorus was the difference between total phosphorus and the sum of the four P fractions extracted above [12]. Total concentrations of Ca, Fe, Mn and Al were determined by the SEPAC method (HJ/T 166-2004) using ICP-AES after wet digestion [47]. The results were calculated on the basis of dry weight sediment.

According to the Unified Soil Classification System (USCS), the sediment particles were classified into four grain sizes: coarse/medium sand fraction (246–840 µm); fine sand fraction (147–246 µm); very fine sand fraction (74–147 µm), and silt/clay fraction (*<*74 µm). The grain size of the sediments was analyzed by an LS 230 laser diffraction particle analyzer (Microtrac S3500).

### Statistical Analysis

Redundancy analysis (RDA) is a multivariate direct gradient analysis that enables the identification of variables that best explain the variance pattern of the P fractions [48], [49], [50]. The data were log (*x*+1) transformed, centered and standardized before a forward selection procedure combined with Monte Carlo permutation tests (499 permutations) were used to identify which factors contributed significantly to the variation (*p*<0.05). The analysis was performed in CANOCO, Version 4 for Windows, and the results are presented in an ordination diagram in which all of the variables are represented by arrows. A smaller angle between arrows represents a high correlation between variables, and the direction of the arrows represents positive or negative correlations. Pearson correlation analyses were used to provide a further quantitative explanation of the correlation between P fractions and metal content. The P fractions contents in the sediments from different sampling sites were subjected to one-way ANOVA to detect significant differences. The correlation and variance analyses were performed in SPSS 18.0.

## Results and Discussion

### The Contents of P Fractions and Related Metals in the Manwan Reservoir

The statistical results with respect to different P fractions, related metals and grain size of the sediment particles of the Manwan Reservoir are presented in Table 2. The contents of different P fractions varied greatly. Ex-P is dissolved P, which is absorbed lightly onto the surface of sediment particles or is released from leached P or CaCO_3_-associated P from organic debris [9], [51]. It can estimate the amounts of phosphorus immediately available. In Manwan Reservoir, ex-P consisted of the minimum part of the P pool (0.1% on average). BD-P is mainly redox sensitive P that binds to Fe-hydroxides and Mn compounds and is considered to be potentially available to algae [9], [28]. Under the anaerobic conditions of the water-sediment interface, BD-P will be released into the water by reductive Fe dissolution. In Manwan Reservoir, BD-P constituted a minor part of total phosphorus (TP) of the sediments (4.9% on average) which was relatively lower than in other reservoirs. Using the same extracting methods in Lake Simcoe, which is the largest lake in South Ontario and is considered mesotrophic (20%–42% TP), Dittrich et al. found that BD-P was the dominant fraction, accounting for 40% and 57%, respectively, of the long- and short-term sediment P release [52]. In Manwan Reservoir, reactive NaOH-P was the third most abundant P fraction in the sediments and accounted for 19.6% of the total phosphorus, on average. Reactive NaOH-P mainly presented as P bound to the surface of aluminium oxides released at high pH levels because of the ligand exchange reactions of hydroxide ions replacing orthophosphate [12], [53], as well as some interior Fe oxides that were not extracted in the BD step [8]. Further, in previous studies, NaOH-P has been used to estimate both short- and long-term available P in the sediments and was verified to be an indicator of algal-available P [54], [55]. HCl-P mainly represents calcium-bound P [56], which appears to be non-motile and is not easily bio-available in the sediments [24], [57], [58]. HCl-P was the most abundant P fraction in the sediments of Manwan Reservoir, constituting 43.6% of TP on average, almost equal to the percentage in Lake Simcoe [52]. Residual-P includes organic phosphorus and refractory P compounds. In Manwan Reservoir, residual-P was the second most abundant P fraction, accounting for 31.9% of TP on average. The rank order of the average concentrations of the four metals in the sediments was Al>Fe>Ca>Mn. The analytical results also showed that there was great spatial variation in the distribution of sediment particles in Manwan Reservoir. According to the average percentage, the silt/clay fraction composed the largest part of all fractions (47.9% on average). The sand fraction, including very fine, fine, and coarse sand, accounted for 45.2% total. Table 2 also shows that Ex-P, Ca, and the coarse sand fraction had relatively high coefficients of variation, which indicated that their contents varied greatly in Manwan Reservoir.

**Table 2:** Statistical analysis of P fraction concentrations, metals in the sediments and grain size of the sediments of the Manwan Reservoir.

	Range (µg/g)	Average percentage (%)	Average concentration (µg/g)	SD	CV
ex-P	0–1.4	0.1	0.5	0.4	0.8
BD-P	8.7–80.4	4.9	35.5	21.7	0.6
NaOH-P	57.3–270.2	19.6	155.3	67.7	0.4
HCl-P	137.5–403.0	43.6	315.6	91.5	0.3
Residual-P	66.2–369.8	31.9	228.8	97.4	0.4
Al	3.89×10^4^–7.57×10^4^	49.4	6.07×10^4^	1.17×10^4^	0.2
Ca	2.37×10^3^–4.61×10^4^	31.6	2.25×10^4^	1.14×10^4^	0.5
Fe	2.65×10^4^–4.31×10^4^	18.4	3.71×10^4^	4.62×10^4^	0.1
Mn	2.71×10^2^–1.09×10^3^	0.6	6.76×10^2^	1.81×10^2^	0.3
S/C	–	47.9	–	33.3	0.7
VFS	–	14.5	–	8.8	0.6
FS	–	12.1	–	15.2	1.3
CS	–	18.6	–	29.9	1.5

S/C: slit/clay fraction (<74 µm).

VFS : very fine sand (74–147 µm).

FS: fine sand (147–246 µm).

CS: coarse sand (246–840 µm).

### Spatial Variation of P Fractions in the Mainstream and Tributaries

**Figure 2:**
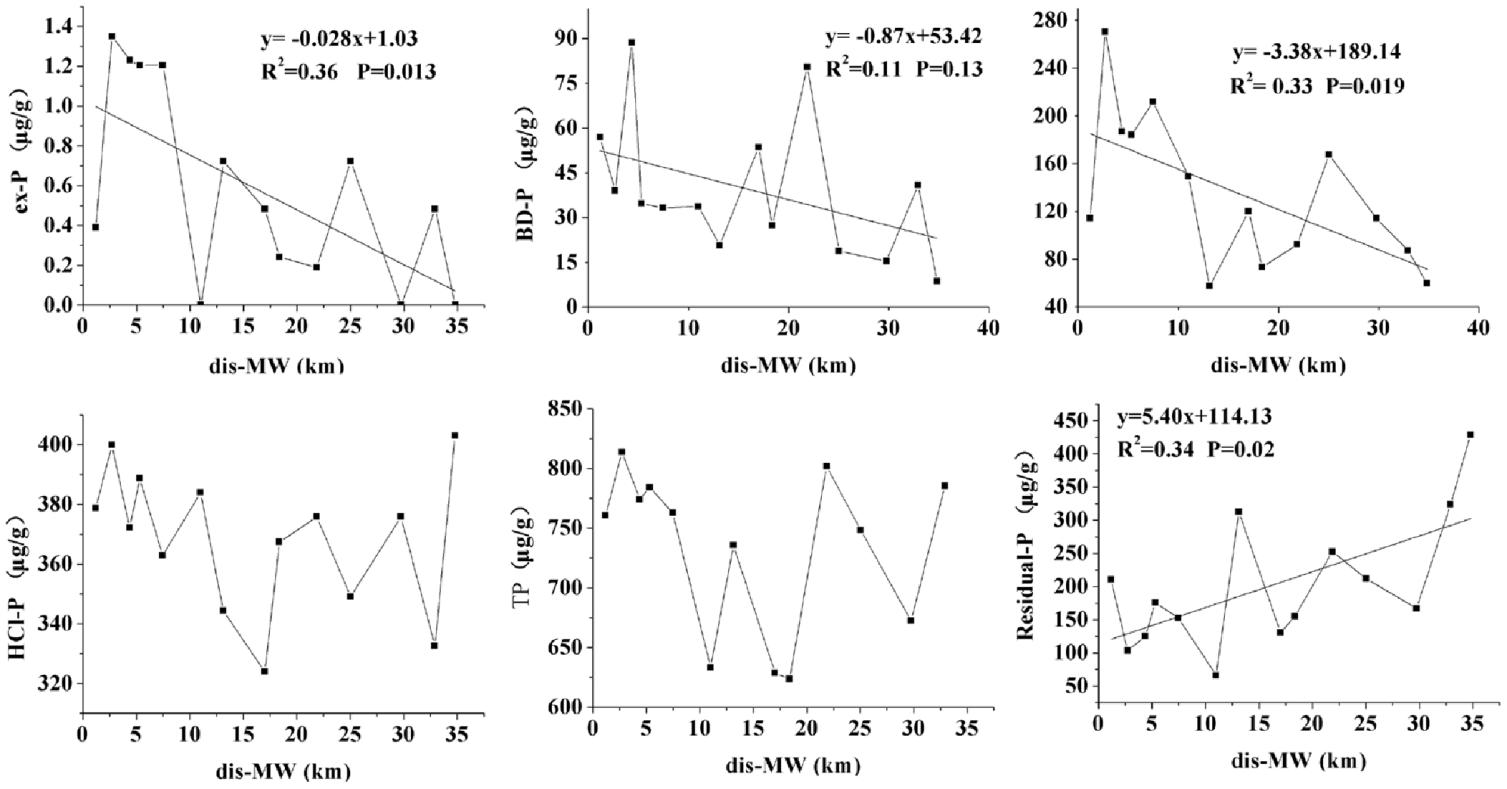
Spatial variation of P fractions in the sediments of the mainstream in Manwan Reservoir.

The relationship between concentrations of P fractions and the distance from the sampling site to Manwan Dam (dis-MW) in the mainstream is presented in Fig. 2. In the mainstream, the changing trends of ex-P, BD-P and NaOH-P were similar from the tail to the head of the Manwan Reservoir. The average content of these three P-fractions increased with decreasing distance to the Manwan Dam. Especially within 9 km from the Manwan Dam, the average content of these three P fractions was relatively higher than it was in other parts of the Manwan Reservoir. Particularly, NaOH-P increased faster than ex-P and BD-P. Unlike ex-P, BD-P and NaOH-P, there was not a consistent pattern in the distribution of HCl-P and TP with respect to the distance to the Manwan Dam; further, there was an upward trend in the concentration of residual-P from Manwan to Xiaowan Dam.

**Figure 3:**
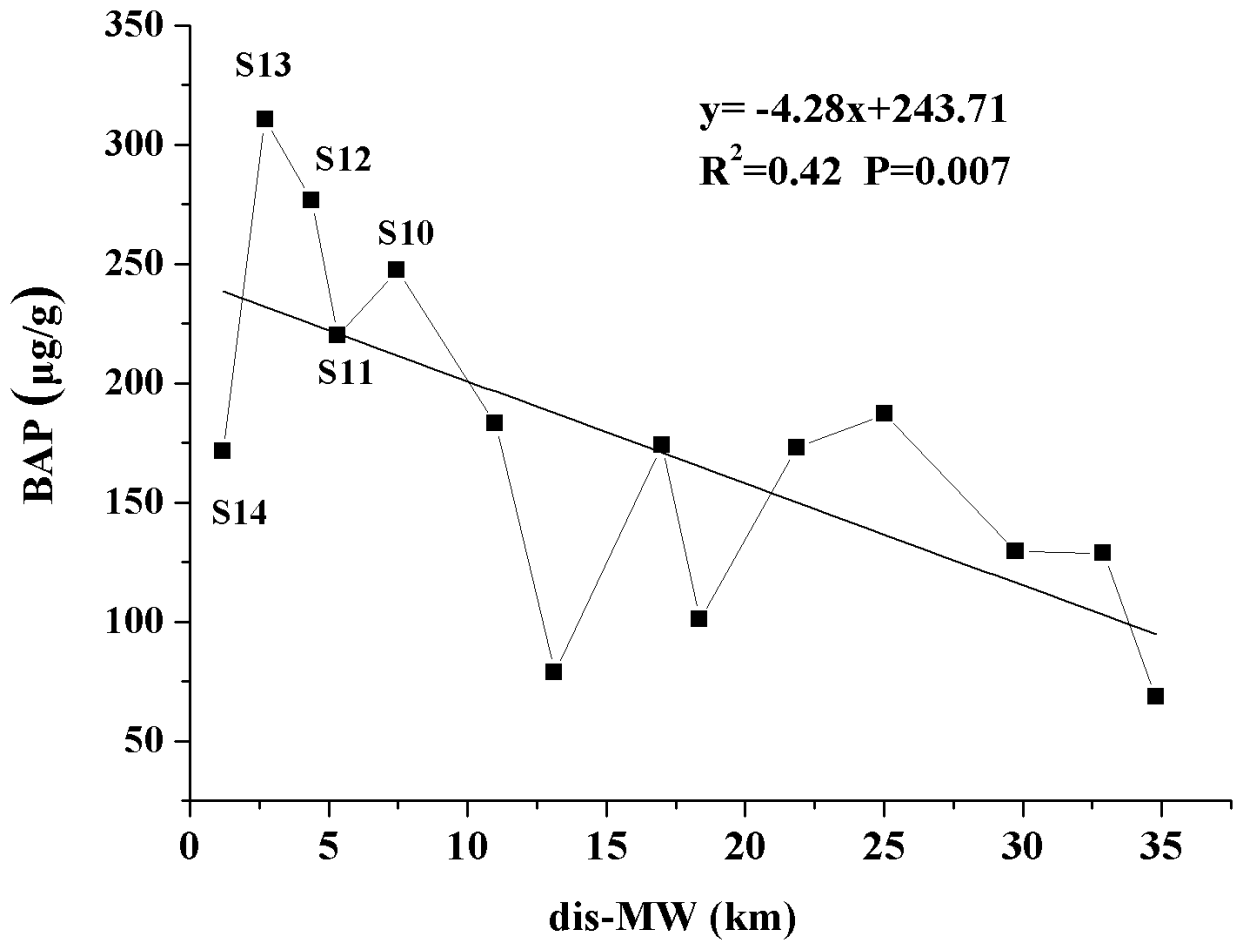
Spatial variation of BAP in the sediments of the mainstream in Manwan Reservoir.

**Figure 4:**
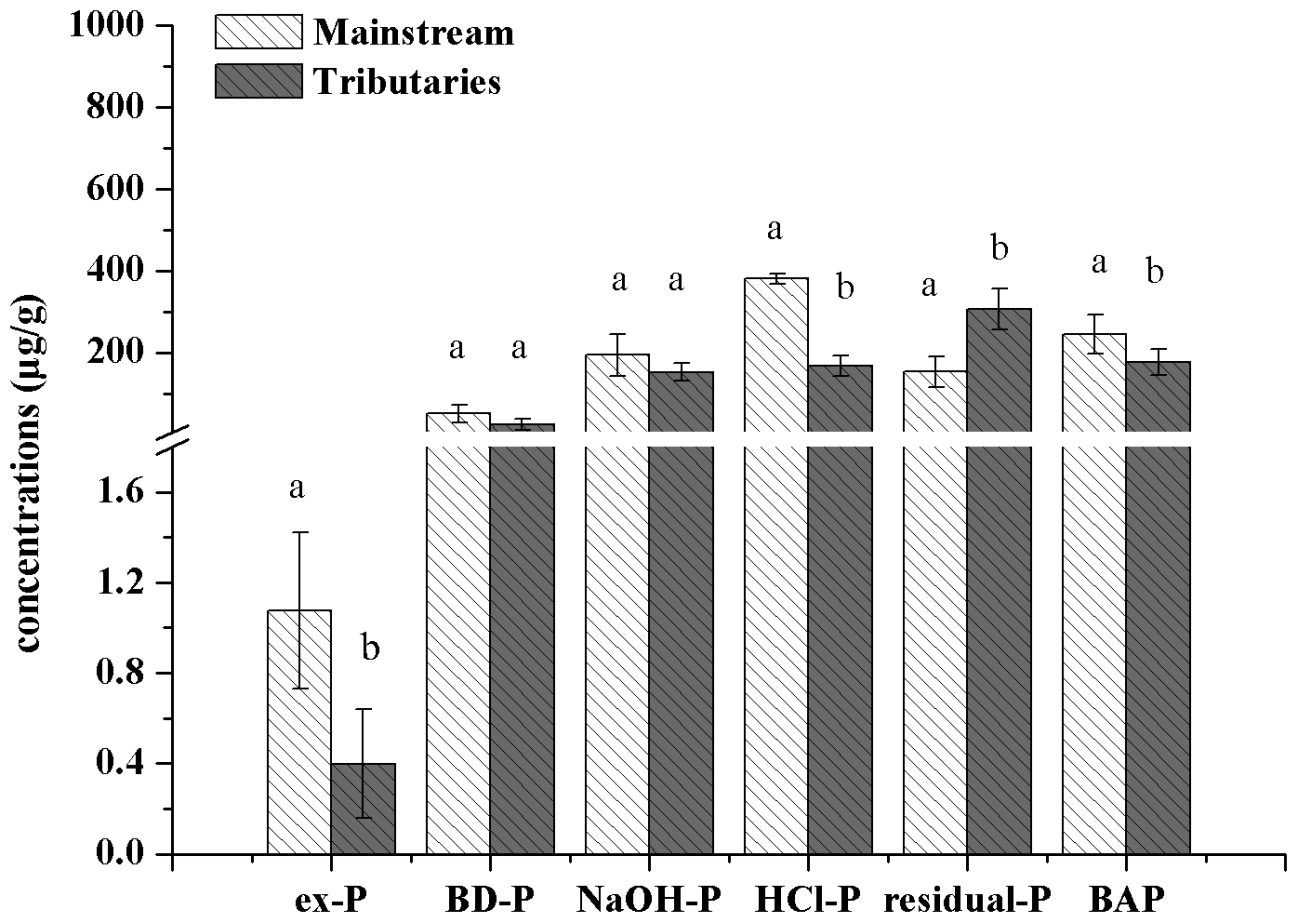
Average concentrations of P fractions in the sediments of mainstream and tributaries. (Different letters indicate significant differences among the mainstream and the tributaries at the 0.05 level).

A previous study has shown that in the Haihe River, China, the amount of P released from sediments was related closely to the ex-P and BD-P fractions, indicating that the P from these two fractions can be released easily [29]. Furthermore, previous studies have estimated the bio-available phosphorus (BAP) in sediments according to the sequential chemical extraction method by the sum of ex-P, BD-P and NaOH-P [21]. By virtue of this theory, Fig. 3 presents the spatial variation of BAP contents in the mainstream of Manwan Reservoir. The values of BAP were at a maximum in the section between S10 and S14, indicating a higher release risk of bio-available P in the sediment. Further, five tributaries were situated within a distance of 10 km from Manwan dam. Samples in the mainstream (S10–S14) and tributaries (S15–S19) within a distance of 10 km to Manwan Dam were selected to compare the spatial variation of P fractions and BAP using a one-way ANOVA (Fig. 4). The results revealed that there were significant differences in the concentrations of ex-P, HCl-P, residual-P and BAP values between the mainstream and the tributaries at the head of Manwan Reservoir. In the mainstream, the average concentrations of ex-P, HCl-P and residual-P were 1.08 µg/g, 380.50 µg/g and 153.47 µg/g, respectively, whereas in the tributaries, the concentrations of ex-P and HCl-P were smaller by 63% and 56%, respectively, than those in the mainstream. Additionally, the average concentration of residual-P was significantly higher in tributaries than in the mainstream. Also in the mainstream, the contents of BAP were significantly higher than those in the tributaries. Because there are more tributaries at the head of Manwan Reservoir, the untreated sewage and drainage of phosphorus from the agricultural land near the tributaries will merge into the mainstream, causing an increase in inorganic P in the sediments.

The total phosphorus (TP) and the proportion of BAP contents in inorganic P (IP) in Manwan Reservoir compared with other freshwater systems is presented in Table 3. The concentration of TP in Manwan Reservoir ranged from 623–899 µg/g, which was lower than other mesotrophic lakes, indicating that Manwan Reservoir may be low-to-mesotrophic. Therefore, phosphorus released by sediments may influence the water quality greatly. The percentage of BAP in IP indicated that the potential risk of P releasing in the Manwan Reservoir was relatively lower than that in other lakes and rivers. However, constant monitoring and further study are still required to understand the future internal loading in Manwan Reservoir.

**Table 3:** Comparison of TP and the proportion of BAP in IP in different surface sediments.

Sediment source	TP(µg/g)	BAP in IP (%)	Trophic classification	Reference
Lake Koronia, Greece	1156–1305	40–60	hypereutrophic	Christophoros Christophoridis et al, 2006
Lake Volvi, Greece	776–1044	60–80	meso-to-eutrophic	Christophoros Christophoridis et al, 2006
Lake Erken, Sweden	1814	61.4	mesotrophic	Emile Rydin, 2000
Haihe River, China	968–2017	42.2–65.3	mesotrophic	SUN Shujuan et al, 2009
Manwan Reservoir	623–899	37.7	–	Present study

### Relationship between P Fractions and Influencing Factors

Table 4 shows the concentrations of Al, Ca, Fe and Mn in sediments both in the mainstream and the tributaries of the Manwan Reservoir. The rank order of the concentrations of these four metals both in mainstream and tributaries was Al>Fe>Ca>Mn. The Al content in the tributaries was higher than that in the mainstream, whereas the content of Fe, Ca and Mn in the tributaries was 20%, 47% and 48% lower, respectively, than those in the mainstream. Al and Fe are lithogenous materials and are conservative in the migration process, which contributes to the high content of these metals in the sediments [35], [59].

**Table 4:** P-related metals concentrations in the Manwan Reservoir (×10^4^ µg/g).

		Al	Ca	Fe	Mn
Mainstream	Range	3.67–7.15	1.55–4.18	2.98–4.31	0.05–0.11
	Mean	5.97	2.43	3.73	0.07
Tributaries	Range	3.89–7.58	0.22–4.61	2.65–4.21	0.03–0.07
	Mean	6.41	1.65	3.65	0.05

Correlation analysis was used to examine the relationship between the contents of P fractions and related metals (Table 5). The results indicated that there were close relationships among P fractions, metals and the grain size fraction of the sediments. Both ex-P and BAP were correlated positively with the contents of the silt/clay fraction in the sediments; BAP and TP were correlated negatively with the contents of the coarse/medium sand fraction (246–840 µm) and the fine sand fraction (147–246 µm). A significant correlation between the concentration of iron and the silt/clay fraction of sediments was also observed (r = 0.544, *p*<0.05). Previous researchers found that grain size has an effect on the chemical composition of the sediments, including the metal contents and the P sorption-desorption ability [25], [55]. As a result, the silt/clay fraction may contain more elements, such as iron, which are very important in adsorbing different P fractions, especially NaOH-P and BD-P compositions of BAP [12], [60], [61]. Ex-P is another fraction of BAP that includes phosphorus lightly absorbed onto the surface of sediment particles; therefore, it is associated closely with the surface physical characteristics of sediments. As the silt/clay fraction has more surface area, so it can absorb more ex-P. The results were consistent with studies performed in the mainstream of Haihe River and Keelung River in China, where a significant correlation between ex-P and silt/clay fraction was found and iron showed a positive linear relationship with fine-grained sediments (grain size<63 µm) [55], [60]. Distance to Manwan Dam was correlated significantly with the contents of NaOH-P and BAP, and was also correlated with the contents of coarse sand (r = 0.581, *p*<0.05) and aluminium (r = −0.486, *p*<0.05) in the sediments (Table 5), indicating that the sediments farther from Manwan Dam contain more coarse sand with fewer aluminium oxides bound to the surface of sediments, which leads to the reduction of NaOH-P and BAP.

**Table 5:** The correlation analysis among P fractions, metals and grain size of sediments.

	ex-P	BD-P	NaOH-P	HCl-P	BAP	TP	S/C	VFS	FS	CS	Al	Ca	Fe	Mn	Dis-MW
ex-P	1														
BD-P		1													
NaOH-P	0.550*		1												
HCl-P				1											
BAP	0.613*		0.942**		1										
TP				0.522*		1									
S/C	0.791**				0.497*		1								
VFS								1							
FS						−0.537*			1						
CS					−0.504*					1					
Al	0.470*										1				
Ca			−0.524*		−0.494*							1			
Fe						0.501*	0.544*	−0.629**	−0.723**	−0.488*	0.584*		1		
Mn	0.536*					0.529*							0.527*	1	
dis-MW			−0.631**		−0.634**					0.581*	−0.486*				1

*p*<0.01;

*p*<0.05.

BAP: bio-available phosphorus FS: fine sand (147–246 µm) CS: coarse sand (246–840 µm).

S/C: slit/clay fraction (<74 µm) VFS : very fine sand (74–147 µm) Dis-Manwan: distance to Manwan Dam.

Flow variability affects the ecological patterns and processes in river systems, such as the nutrients dynamic [26]. Dams can manipulate the flow regime and consequently, in the upstream of the dam, fine suspended particles are captured and accumulated from the floodplain, while the downstream channel becomes eroded, leading to the coarsening of the sediments [62], [63], [64] Because of the combined effect of Xiaowan and Manwan dams, the flow velocity downstream from Xiaowan Dam is much higher than in the upstream of Manwan Dam, leading to coarser sediments fractions downstream of Xiaowan Dam. As it approaches the Manwan Dam, the river surface widens and the flow velocity slows, resulting in the accumulation of more slit/clay sediment fractions. According to correlation analysis, the contents of total phosphorus (TP) were correlated positively with Fe (r = 0.501, *p*<0.05). Previous studies found that there was an apparent relationship between TP and Fe [12], [44]. In the Manwan Reservoir, the HCl-P content was not related to Ca. This finding is consistent with other studies [43], [55], [65], and can be attributed to the different sources of HCl-P, including sedimentary processes, the exchanges between calcium- and iron-bound phosphate [66] and P from fertilizer caused by runoff [27], [43].

**Figure 5:**
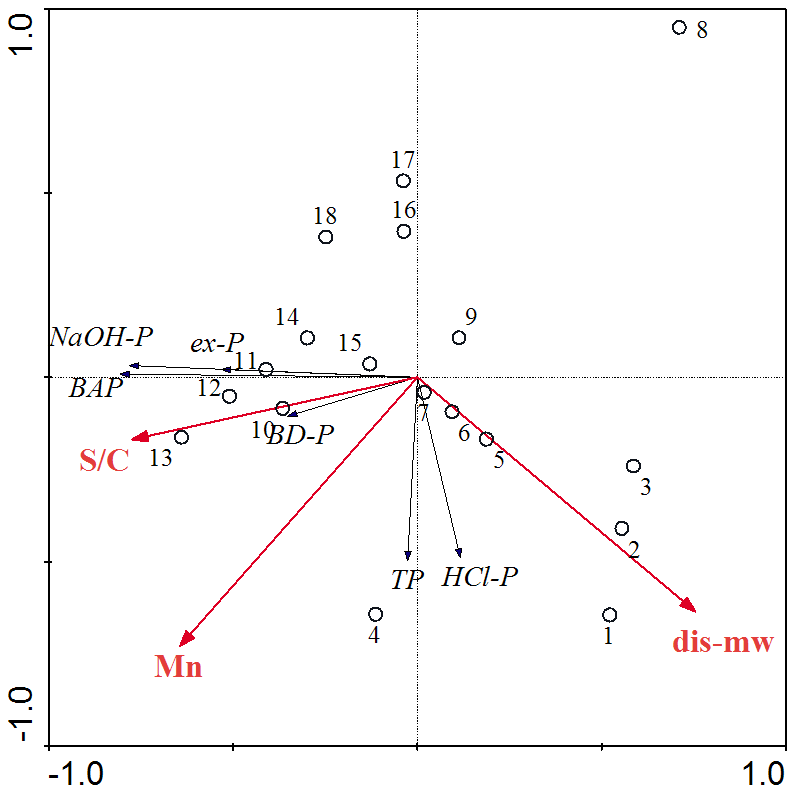
RDA results for P fractions, metals and grain size of sediments.

Redundancy analysis (RDA) was performed, using four P fractions, bio-available P, and total P as response variables and using metals, distance to Manwan hydropower station and grain size distribution as explanatory variables. The percentage of silt/clay fraction in sediments, distance to Manwan hydropower station and the concentration of Mn in sediments explained the variation significantly (*p* = 0.014, 0.018 and 0.05, respectively; Fig. 5). These three variables accounted for 54% of the total variance, of which the percentage of silt/clay fraction accounted for 20%, distance to Manwan hydropower station explained 19% and the concentration of Mn explained 15%. The percentage of silt/clay fraction correlated positively with the contents of ex-P, BD-P, NaOH-P and BAP in sediments, and the contents of Mn correlated positively with BD-P. RDA analysis also revealed the relationship between P fractions and other factors. Further, RDA can present the relationship between different sampling sites. Concentrations of NaOH-P were correlated positively with ex-P, which was mainly due to its association with the silt/clay fraction of the sediments. The samples from the study sites were distributed in different quarters of the RDA biplot. Samples in the mainstream at the head of the Manwan Reservoir (S10–S14) were related to ex-P, BD-P, BAP and silt/clay fraction of the sediments, which represented a high release risk of P.

## Conclusions

Because of the construction of dams, reservoirs have become sensitive regions that have attracted more and more attention in recent years. The release of phosphorus from sediments can influence water quality and water purification capacity, which will endanger the beneficial uses of the river [67]. Thus, spatial analysis of P fractions is important in understanding nutrient pollution and potential ecological risks in the reservoir system. The results of our study are summarized below.

In the mainstream, the rank order of P fractions was HCl-P>NaOH-P>residual-P>BD-P>ex-P. Further, the concentration of ex-P, BD-P and NaOH-P showed an upward trend from Xiaowan to Manwan dam, where the contents of bio-available P (BAP)–the sum of ex-P, BD-P and NaOH-P–were relatively higher from S10 to S14 in the mainstream, especially within approximately 10 km of the Manwan Dam. Moreover, the concentration of BAP in the mainstream was significantly higher than it was in the tributaries; this indicated a greater release risk of phosphorus and more immediately available P in the water. In contrast, there was no trend in the contents of HCl-P and total phosphorus (TP) along the longitudinal direction of Manwan Reservoir. Hence, it may be better to estimate the internal phosphorus loading of the Manwan Reservoir by the contents of BAP rather than TP in the sediments.

In this study, correlation and redundancy analyses (RDA) revealed that silt/clay fraction contents of sediments and distance to Manwan Reservoir influenced the spatial variation of P fractions strongly, especially the bio-available fractions including ex-P, BD-P and NaOH-P. The contents of total phosphorus in the sediments of Manwan Reservoir was 623–899 µg/g, which were lower than some mesotrophic lakes in the world and depended primarily on the contents of iron in the sediments.

The techniques used in this study can be employed widely in freshwater systems and can contribute to the investigation of the tropic conditions, as well as evaluate the potentially mobile P pool stored in canyon reservoir sediments. Especially for an international river like the Mekong nearly half the length of which was in Yunnan Province in China, the construction and operation of cascade dams upstream may cause significant ecological risks downstream [68], risks that are of international concern. Our studies demonstrated that there exist “longitudinal effects” of dams on some phosphorus fractions. Therefore, a management plan was recommended that will intensify the monitoring of the internal P loading of the reservoir, as well as reduce the external P loading that includes the non-point loading from tributaries.
